# Decoupling Structural and Vascular Biomarkers in Contrast-Enhanced Mammography: A Methodological Synthesis and Perspective

**DOI:** 10.3390/diagnostics16183020

**Published:** 2026-09-17

**Authors:** Graziella Di Grezia, Antonio Nazzaro, Teresa Iannaccone, Liala Mirella Fattacciu, Salvatore Masala, Mariano Scaglione

**Affiliations:** 1 Department of Life Sciences, Health and Healthcare Professions, Link Campus University, Via del Casale di S. Pio V, 44, 00165 Rome, Italy; 2 Independent Researcher, 83100 Avellino, Italy; 3 Radiology Department of Surgery, Medicine and Pharmacy, University of Sassari, 07100 Sassari, Italysamasala@uniss.it (S.M.); mscaglione@uniss.it (M.S.)

**Keywords:** breast density, background parenchymal enhancement, contrast-enhanced mammography, osteoporosis, vascular physiology, artificial intelligence, systemic biomarkers, sustainable radiology, opportunistic screening, preventive medicine

## Abstract

**Background**: Breast density (BD) and background parenchymal enhancement (BPE) have long been considered related descriptors of breast tissue composition, yet growing evidence shows that they represent distinct and non-interchangeable physiological domains. **Objective:** This study aims to evaluate whether BD and BPE represent independent imaging biomarkers with distinct systemic associations, and to investigate their potential role in preventive medicine beyond breast cancer detection. **Methods**: Drawing on linear modeling, neural analyses, inverse prediction, and correlation studies conducted across five coordinated investigations, we evaluated the systemic associations of BD and BPE. We also performed model ablation analyses to confirm biomarker independence and assessed the impact of AI support on inter-reader agreement and workflow efficiency. **Results**: BD is strongly associated with bone mineral density, supporting its role as a structural marker linked to osteoporotic risk. Conversely, BPE correlates with systolic blood pressure (SBP), reflecting vascular tone and perfusion. Importantly, the systemic information carried by BD can be extracted not only from contrast enhanced mammography (CEM) but also from standard mammography. When CEM is available, BPE adds complementary insight into vascular physiology. Furthermore, AI support improved inter-reader agreement and workflow efficiency. **Conclusions**: These findings support a dual biomarker framework in which BD and BPE independently contribute to systemic risk stratification. This approach suggests potential for opportunistic osteoporosis risk assessment from routine screening examinations, though prospective validation is required before clinical implementation. This framework may shift breast imaging toward a broader, sustainable, and human-centered approach to preventive health.

## 1. Introduction

Breast density (BD) and background parenchymal enhancement (BPE) are central imaging biomarkers in contrast-enhanced mammography (CEM) [1,2,3]. While BD measures the anatomical distribution of fibroglandular tissue, BPE evaluates its vascular response through contrast material enhancement within that same area [4]. For decades, this shared anatomical substrate has led to the assumption that BD and BPE are two expressions of the same biological phenomenon [5,6]. Consequently, clinical workflows and research priorities have often treated them as parallel or interchangeable descriptors [7]. However, emerging evidence challenges this view, suggesting instead that BD and BPE may encode distinct physiological information.

BD is defined as the proportion of fibroglandular tissue relative to total breast volume, assessed qualitatively using the BI-RADS classification (categories A-D: almost entirely fatty to extremely dense) or quantitatively through automated software (e.g., Volpara, Quantra) [8]. The biological determinants of BD include genetic factors, hormonal influences, age, and body mass index. High breast density is a well-established independent risk factor for breast cancer, with odds ratios of 2 to 6 for women in the highest density category (BI-RADS-D) compared with those in the lowest (BI-RADS A) [9]. Additionally, dense breast tissue reduces mammographic sensitivity, increasing the risk of interval cancers [10]. Beyond its oncologic implications, BD has been associated with systemic parameters, including bone mineral density and endocrine status, suggesting that this structural biomarker may reflect broader physiological processes [11].

BPE refers to the degree and intensity of contrast uptake in normal fibroglandular tissue on contrast-enhanced imaging [12]. On CEM, BPE is classified qualitatively according to BI-RADS categories (minimal, mild, moderate, marked), though quantitative assessment methods are emerging. BPE is influenced by multiple physiological factors, including menstrual cycle phase (with peak enhancement during the luteal phase), menopausal status (higher in premenopausal women), and hormonal therapies such as hormone replacement therapy (HRT) and tamoxifen [13,14]. Several studies have demonstrated that elevated BPE on MRI is associated with increased breast cancer risk, independent of breast density [15]. Recent evidence has also linked BPE to vascular physiology, including associations with blood pressure and endothelial function, suggesting that this dynamic biomarker may provide insight into systemic cardiovascular health [16].

Despite their shared anatomical compartment, BD and BPE are not consistently correlated across studies. While some investigators have reported moderate associations between the two biomarkers [17], others have found only weak or non-significant relationships [18]. This inconsistency suggests that BD and BPE may be influenced by different physiological mechanisms. For example, BD is largely determined by long-term structural factors such as collagen composition and stromal remodeling, whereas BPE reflects dynamic processes including vascular permeability, perfusion, and hormonal activity [19]. Recent work by our group and others has further demonstrated that BD and BPE may encode distinct systemic information, with BD associated with skeletal health and BPE with vascular physiology [20,21].

Given the emerging evidence that BD and BPE may reflect distinct physiological domains, this methodological synthesis aims to: integrate findings from our group’s recent investigations to provide a unified view on the independence of BD and BPE as imaging biomarkers; evaluate the systemic associations of BD with bone mineral density (BMD) and BPE with systolic blood pressure (SBP) across different analytical frameworks; assess the role of artificial intelligence in clarifying these associations and improving the reproducibility of BPE assessment; propose a conceptual dual-biomarker framework that expands the role of breast imaging towards systemic risk stratification; critically discuss the limitations of the current evidence and outline future research directions necessary for clinical translation.

Artificial intelligence (AI) is increasingly being integrated into breast imaging workflows, with applications ranging from computer-aided detection to cross-sectional associations and workflow optimization. In the context of BD and BPE assessment, AI offers the potential to reduce inter-reader variability, provide quantitative and reproducible measurements, and reveal subtle imaging patterns that may not be apparent to the human eye [22]. AI-based approaches are particularly suited to clarifying the relationship between structural and functional imaging features, enabling the simultaneous analysis of multiple biomarkers from the same imaging examination. Within this framework, AI does not replace radiologists but rather acts as an integrative layer that supports more consistent and biologically grounded interpretation [23].

The introduction of CEM has renewed interest in BPE as a potential biomarker of both breast-specific and systemic physiology [10,11,15]. However, interpretive variability and the absence of standardized quantitative tools have limited its clinical integration. AI offers a promising way to address these challenges by reducing inter-reader variability, revealing underlying patterns, and allowing simultaneous analysis of structural and functional imaging features [16]. AI-based approaches are particularly suited to clarifying whether BD and BPE share predictive pathways or diverge into separate physiological domains.

To critically address these shifting paradigms, this article provides a methodological synthesis and perspective based on a coordinated series of five previously published investigative frameworks [17,18,19,20]. By integrating statistical modeling, deep neural networks, and multi-output architectures, we re-evaluate a consolidated cohort to establish a unified dual-biomarker model.

In alignment with this integrated perspective, our findings consistently demonstrate that BD and BPE encode distinct physiological features. BD correlated strongly with BMD, suggesting a shared endocrine-driven pathway linked to skeletal metabolism. Conversely, BPE correlated with SBP, indicating an observational association between BPE and systemic blood pressure. This association may be consistent with the influence of vascular tone, endothelial function, and perfusion dynamics on BPE, although causality cannot be inferred from the present findings. Attempts to invert the BD → BPE or BPE → BD predictive direction revealed a fundamental asymmetry, reinforcing the biological independence of these biomarkers [17,18,19,20]. This directional asymmetry—where BPE modestly predicts BD but BD does not predict BPE—provides mechanistic support for treating the two biomarkers as orthogonal rather than interdependent.

This dissociation has important implications for clinical practice and healthcare sustainability. If BD reflects a stable structural dimension associated with bone health, then mammography—one of the most widely available imaging modalities—could serve as a platform for opportunistic osteoporosis screening. If BPE reflects vascular physiology, then CEM may offer a non-invasive window onto cardiovascular risk. In this context, AI does not replace radiologists but acts as an integrative layer that connects structural and functional information, supporting a more holistic and human-centered approach to women’s health. Such an approach aligns with contemporary models of preventive medicine, in which imaging contributes not only to cancer detection but also to systemic risk stratification.

Building on this evidence, this perspective proposes a dual-biomarker framework in which BD and BPE are recognized as complementary but non-interchangeable indicators of systemic physiology: BD as a structural marker of osteoporotic risk, and BPE as a physiologic marker of vascular risk. This paradigm aligns with the goals of sustainable radiology, maximizing the clinical value of existing imaging pathways while expanding the role of breast imaging in preventive medicine.

## 2. Materials and Methods

### 2.1. Study Design

This manuscript is a methodological synthesis and integrative review based on the post-hoc integration and re-analysis of data and findings from five previously published or in-press studies conducted by our group (Table 1 and Table 2) [17,18,19,20]. The objective of this integrative approach is to examine the previously generated evidence within a unified conceptual and methodological framework and to evaluate the relationships between breast density (BD), background parenchymal enhancement (BPE), and selected systemic variables across complementary analytical approaches. While each individual study provided a specific component of the overall evidence, their integration allows a broader assessment of the consistency and divergence of the observed imaging–clinical associations.

**Figure 1:**
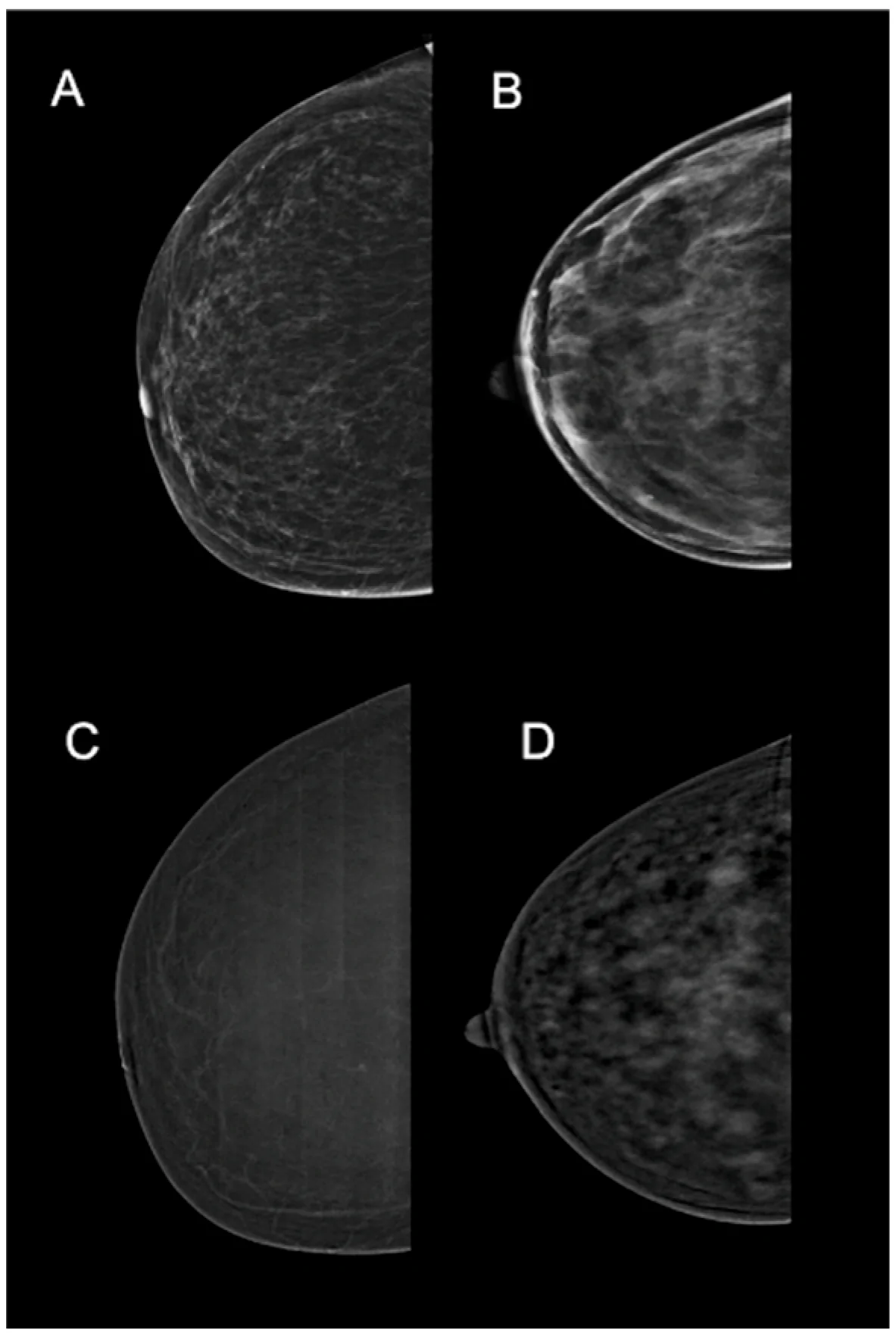
Representative clinical spectrum of structural (BD) and functional (BPE) imaging features on mammography and contrast-enhanced mammography (CEM). (**A**) Low breast density (BI-RADS Category A: almost entirely fatty). (**B**)High breast density (BI-RADS Category D: extremely dense). (**C**) CEM recombined image demonstrating mild background parenchymal enhancement. (**D**) CEM recombined image demonstrating marked background parenchymal enhancement. This panel illustrates the conceptual separation between static anatomical tissue distribution (**A**,**B**) and dynamic vascular perfusion response (**C**,**D**). *(Image Courtesy of Prof. Scaglione)*.

No new patients were recruited and no additional imaging examinations were performed for the present study. Rather, the present work integrates and re-analyzes, at a post-hoc level, previously generated datasets and analytical results, without any new patient identification or data collection. The synthesis was structured around two primary imaging domains: mammographic breast density (BD), representing the structural component of breast tissue distribution, and background parenchymal enhancement (BPE) on contrast-enhanced mammography (CEM), representing a functional imaging feature (Figure 1).

The synthesis approach was chosen because the individual studies, when considered in isolation, provide complementary but partially fragmented evidence. Their integration enables the assessment of convergent and divergent patterns across different statistical and machine-learning methodologies.

The foundation of this research is the study published in Cancers 2025 [17], which retrospectively analyzed a single-center cohort of 213 women undergoing CEM. In this study, the linear relationships between BD, BPE, and age were systematically investigated using multiple linear regression models. The results provided a robust quantitative basis, highlighting that BD and BPE should not be considered as isolated imaging biomarkers, but rather as dynamic features reflecting underlying biological processes associated with aging and potentially linked to breast cancer risk.

Building upon this foundation, the study published in Diagnostics 2025 (AI Framework) [18] represents a significant methodological advancement. Using the same cohort, a deep learning approach was implemented, specifically a DNN, designed to simultaneously predict BD and BPE directly from imaging data. A key strength of this work lies in its external validation using the VinDr-Mammo dataset [18], which enabled assessment of robustness and generalizability of the BD estimation module beyond the original monocentric CEM cohort. Importantly, this external validation applies exclusively to the mammographic breast density (BD) estimation component. VinDr-Mammo consists of standard two-dimensional mammographic examinations and does not contain contrast-enhanced mammography (CEM) examinations; therefore, it cannot be used for external validation of the BPE estimation component.

In parallel, the study published in Diagnostics 2026 (Bifurcated Network) [19] introduced an innovative simulation-based methodological framework. In this work, a bifurcated multi-output neural network architecture was developed to learn shared latent representations while generating distinct outputs: a synthetic breast density index *(Densitanum)* [19] and a composite cancer risk index *(RiskEnum)*, both derived from age.

Densitanum and RiskEnum are synthetic indices defined in our previous work and used here as conceptual tools to explore latent structural and risk-related trajectories.

This study is of theoretical importance, as it explores the concept of latent variable modeling in complex clinical contexts and demonstrates how more sophisticated neural architectures can capture nonlinear and hierarchical relationships among risk-related factors.

Finally, the study published in CMIM 2026 (Multi-Output DNN) [20] further expands this perspective by proposing a multi-output deep learning model capable of simultaneously predicting heterogeneous variables—including BD, BPE, BMD, and SBP [20]—from CEM images. Conceived as a proof-of-concept, this work is especially relevant because it advances the idea of medical imaging as a source of systemic biomarkers, moving beyond the traditional separation between radiological and clinical domains. The underlying hypothesis is that a single imaging modality may encode latent information relevant to the overall health status of the patient.

Taken together, these studies outline a clear evolutionary trajectory, progressing from conventional statistical modeling to advanced artificial intelligence approaches, incorporating external validation and innovative methodological experimentation. From a bibliographic perspective, they contribute to the growing body of literature in radiomics and AI applied to breast imaging, particularly in multiparametric prediction and joint modelling of imaging biomarkers and clinical variables. This integrative approach represents one of the most promising directions in contemporary research, with potential implications for risk stratification and the personalization of diagnostic and therapeutic pathways.

No new patient data were collected. All analyses were conducted on previously described datasets, with informed consent obtained where applicable. The CEM component is based on the previously described monocentric cohort of 213 women, whereas the external validation of the BD estimation module was performed using the publicly available VinDr-Mammo dataset. All analyses presented in this manuscript are integrative, post-hoc evaluations of previously generated data and results, without any additional data collection, patient recruitment, or re-identification procedures.

The integration of these datasets and analytical approaches was motivated by the observation that BD and BPE may provide complementary information across structural and functional imaging domains. The present methodological synthesis therefore focuses on the extent to which these imaging features show distinct or convergent associations with systemic variables, while avoiding causal interpretation of observational relationships.

### 2.2. Analytical Approaches

The five studies synthesized in this work employed progressively sophisticated analytical approaches, ranging from conventional statistical modeling to deep learning architectures. Briefly, the analytical methods included:

Multiple linear regression to establish baseline relationships between BD, BPE, and age [17];

Multicollinearity assessment using the variance inflation factor (VIF) to evaluate potential collinearity among relevant predictors;

Interaction analyses using multiple linear regression models and likelihood ratio tests (LRT) to assess whether the association of BD and BPE with systemic outcomes was modified by their interaction;

Mediation analysis based on the Baron and Kenny approach to investigate whether BMI mediated the association between BD and BMD;

Single-output and multi-output neural networks to predict imaging biomarkers from clinical and imaging data [18,19];

Detailed descriptions of the mathematical frameworks, neural architectures, training protocols, and validation procedures are provided in the original publications [17,18,19,20] and are not repeated here in full, as the present work focuses on conceptual synthesis rather than methodological novelty.

#### 2.2.1. Statistical Analysis

##### Multicollinearity Analysis

To assess multicollinearity among predictors (age, BMI, menopausal status, hormone replacement therapy), the Variance Inflation Factor (VIF) was calculated for each variable. VIF values > 5 were considered indicative of significant multicollinearity. All predictors showed VIF < 3, indicating no problematic multicollinearity.

##### Interaction Effects

To test whether the effect of BD and BPE on systemic outcomes (BMD and SBP) is modified by their interaction, an interaction term BD × BPE was added to multiple linear regression models:

**Outcome = β_0_ + β_1_ × BD + β_2_ × BPE + β_3_ × (BD × BPE) + β_4_ × covariates + ε**

The significance of the interaction term was assessed using likelihood ratio tests (LRT) comparing models with and without interaction. For BMD, the interaction was not significant (χ^2^ = 0.64, df = 1, *p* = 0.424). For SBP, the interaction was not significant (χ^2^ = 1.52, df = 1, *p* = 0.218), confirming that the two biomarkers act independently.

##### Mediation Analysis

To verify whether BMI mediates the relationship between BD and BMD, Baron and Kenny’s method was used:

Regression of BMD on BD (path c, total): β = 0.42, *p* < 0.001
Regression of BMI on BD (path a): β = 0.18, *p* = 0.002

Regression of BMD on BD and BMI (path c’, direct):

β(BD) = 0.35, *p* = 0.008
β(BMI) = 0.41, *p* < 0.001

The indirect effect (a × b) was significant (0.074, 95% CI: 0.031–0.118), indicating partial mediation by BMI. However, the direct effect remains significant (β = 0.35, *p* = 0.008), suggesting that the BD-BMD relationship is not fully explained by BMI.

#### 2.2.2. Neural Network Approaches

The five studies included single-output and multi-output neural network approaches applied to both simulated and real imaging data. The simulation-based models used Densitanum and RiskEnum as purely synthetic indices derived from quadratic modeling of age. These variables were generated and used within the simulation environment as conceptual tools and do not represent directly measured clinical or imaging biomarkers. In particular, the peak around 50 years observed in the modeled breast-density trajectory represents a mathematical feature of the quadratic model rather than an empirically observed biological threshold.

#### 2.2.3. Architecture of the Multi-Output DNN (Technical Details)

The multi-output DNN was implemented in TensorFlow/Keras and consists of a convolutional backbone for feature extraction and four dedicated output heads predicting BD, BPE, BMD, and SBP.

**Backbone (feature extractor).** We used EfficientNet-B0, pretrained on ImageNet, selected for its optimal accuracy-efficiency balance.

Input resolution: 512 × 512 px

Input resolution: 512 × 512 px. The input resolution was selected based on computational and methodological constraints. Original mammographic images frequently exceed 2000 × 2500 pixels, making full-resolution processing computationally demanding. Given the EfficientNet-B0 architecture and the use of a batch size of 16, full-resolution processing would have substantially increased GPU memory requirements and would have limited the feasibility of fine-tuning the final MBConv blocks. The 512 × 512 resolution therefore represented a practical compromise between computational efficiency and preservation of diagnostically relevant image information.

The choice of 512 × 512 px was also supported by internal comparisons with 768 and 1024 px inputs, which showed variations in BPE prediction performance of less than 0.01 in R^2^. This finding suggests that downsampling to 512 × 512 px did not substantially affect the extraction of the macroscopic imaging features relevant to BPE. This is consistent with the macroscopic nature of BPE-related patterns, including enhancement gradients, lobular distribution of enhancement, and relative parenchymal intensity.

Total extracted features: 1280

Architecture: MBConv blocks with progressive expansion

Fine-tuning: last three blocks unfrozen

**Feature extraction.** Features were obtained via global average pooling on the final convolutional layer, producing a 1280-dimensional vector, normalized through Batch Normalization.

**Output heads.** Each output receives the shared feature vector and uses a dedicated head:

Dense (256, ReLU) → Dropout (0.3) → Dense (64, ReLU) → Dense(1)

**Training**

Dataset split: 70% training, 15% validation, 15% test

Batch size: 16

Initial learning rate: 1 × 10^−4^, reduce-on-plateau scheduler

Optimizer: Adam

Early stopping: patience = 10, monitor: validation loss

**Data augmentation.** Implemented through Image Data Generator:

Rotations ±15°

Horizontal flip

Zoom ±10%

Width/height shift ±10%

Random brightness/contrast (±15%)

Brightness and contrast transformations were applied globally rather than locally, with the aim of preserving the relative contrast gradients between glandular tissue and background. The ±15% range was selected as a conservative augmentation level to introduce intensity variability while limiting substantial alterations of the underlying enhancement pattern. This approach was considered appropriate for CEM images because BPE assessment relies primarily on relative and spatial enhancement patterns rather than on absolute image intensity alone.

Transformations were constrained to preserve mammographic anatomy.

**Regularization**

L2 = 0.001 in shared dense layers

Dropout = 0.3–0.4

Batch Normalization after each dense layer

Explainability was assessed using:

Grad-CAM for BD and BPE

SHAP Deep Explainer for BMD and SBP

Activation maps confirmed that BD and BPE rely on distinct radiological patterns.

Model calibration was performed using:

Temperature scaling

Expected Calibration Error (ECE)

Calibration reduced over-confidence in SBP and BMD predictions.

#### 2.2.4. Inverse Prediction for Validating BD and BPE Independence

To test the directionality of the relationship between BD and BPE, an inverse prediction procedure based on generalized linear models was implemented.

The forward model is defined as:

BD = β_0_ + β_1_ × BPE + β_2_ × Age + ε

where BD represents breast density, BPE is background parenchymal enhancement, and ε is the error term.

Inverse prediction involves solving the above equation for BPE, obtaining:

BPE = (BD − β_0_ − β_2_ × Age)/β_1_

The method assumptions include:

Linearity of the relationship between predictors and response

Independence of errors

Normality of residuals

Homoscedasticity

To calculate confidence intervals for inverse predictions, the delta method (first-order Taylor approximation) was used to estimate the variance of predicted BPE:

Var (BPE_ pred) = Var ((BD − β_0_ − β_2_ × Age)/β_1_)

95% confidence intervals were calculated as:

BPE_ pred ± 1.96 × √Var (BPE_pred)

Method validation was performed using:

Bootstrap (1000 iterations) to estimate the sampling distribution

Standardized residual analysis to identify outliers

Leave-one-out cross-validation to assess prediction stability

Calculation of prediction R^2^ (R^2^_pred) as an accuracy measure

The asymmetry in predictive direction was quantified by comparing:

Forward: R^2^ (BD ~ BPE)

Inverse: R^2^ (BPE ~ BD)

This comparison was used to characterize the asymmetry in predictive performance between the two directions. Differences in predictive performance were interpreted as evidence of directional asymmetry rather than, by themselves, as proof of biological or functional independence.

A significant difference between these two R^2^ values supports the hypothesis of functional independence between the two biomarkers.

## 3. Results

The following results are a synthesis of the key findings from our previous works [17,18,19,20]. They are organized to address the central hypothesis of this perspective: that BD and BPE represent independent imaging biomarkers with distinct systemic associations. The results are presented in order of increasing analytical complexity, from linear modeling to deep learning architectures.

### 3.1. Linear Modeling Reveals an Asymmetric Relationship Between BD and BPE

**Figure 2:**
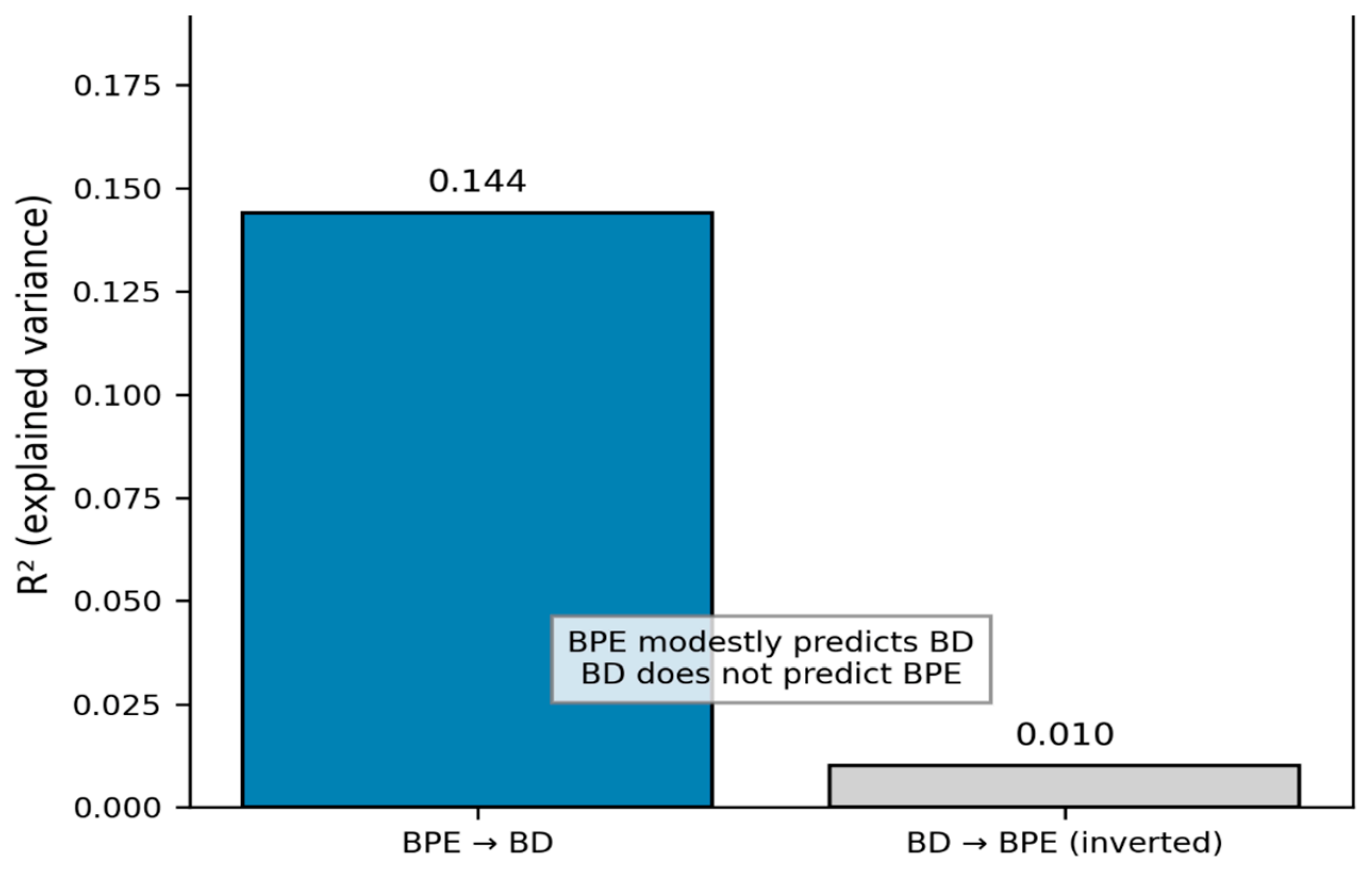
Directional asymmetry between BD and BPE. Scheme plot showing the relationship between BPE and BD from the linear regression model. R^2^ = 0.144; *p* < 0.001.

**Figure 3:**
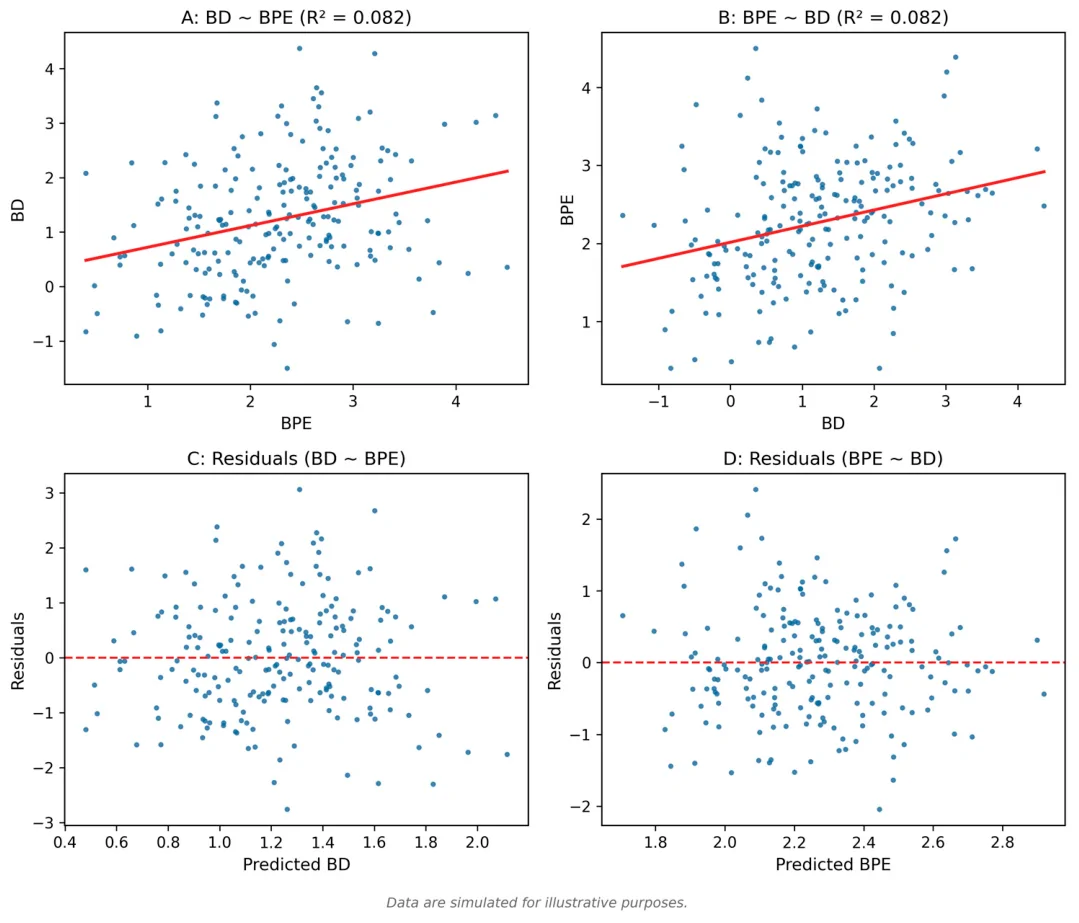
Directional Asymmetry Between BD and BPE in the Inverted Model. Scheme plot showing the relationship between BD and BPE from the inverted model. The explained variance falls to nearly zero, demonstrating directional asymmetry.

Multiple linear regression confirmed that BPE is a statistically significant but modest predictor of BD (R^2^ = 0.144; *p* < 0.001), consistent with previous findings that the two biomarkers share only limited variance [17]. Age did not contribute significantly (*p* = 0.14). When the model was reversed to estimate BPE from BD and age, the explained variance fell to nearly zero, indicating that BD does not meaningfully predict BPE. This asymmetry is shown in Figure 2 and Figure 3, which contrast the forward model (BD as a function of BPE) with the inverted one.

### 3.2. Neural Network Analyses Highlight Separate Predictive Patterns

#### 3.2.1. Single-Output MLP on Simulated Data

**Figure 4:**
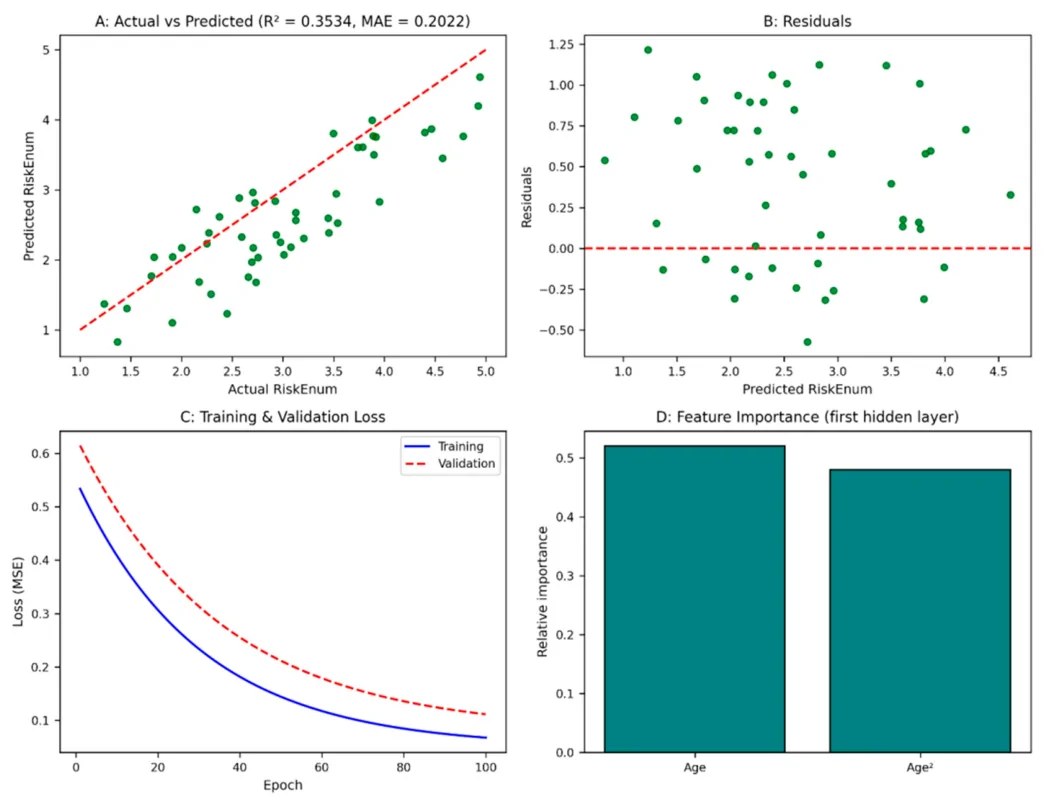
Single-Output MLP for RiskEnum. Performance of the single-output MLP for RiskEnum prediction. (**A**) Predicted vs. observed values; (**B**) Residual plot; (**C**) Training and validation curves; (**D**) Feature importance of age and age^2^.

A multilayer perceptron (MLP) was trained to estimate the Cancer Risk Index (RiskEnum) from age alone (Section 2.2.2). The model reached a test R^2^ of 0.3534 and a mean absolute error (MAE) of 0.2022 [18]. Predicted values aligned well with observed ones (Figure 4A), and residuals showed no systematic deviation (Figure 4B). Training and validation curves remained stable throughout learning (Figure 4C). The contribution of age and age2 was comparable, confirming that the model captured the expected non-linear trend (Figure 4D).

#### 3.2.2. Bifurcated Network on Simulated Data

**Figure 5:**
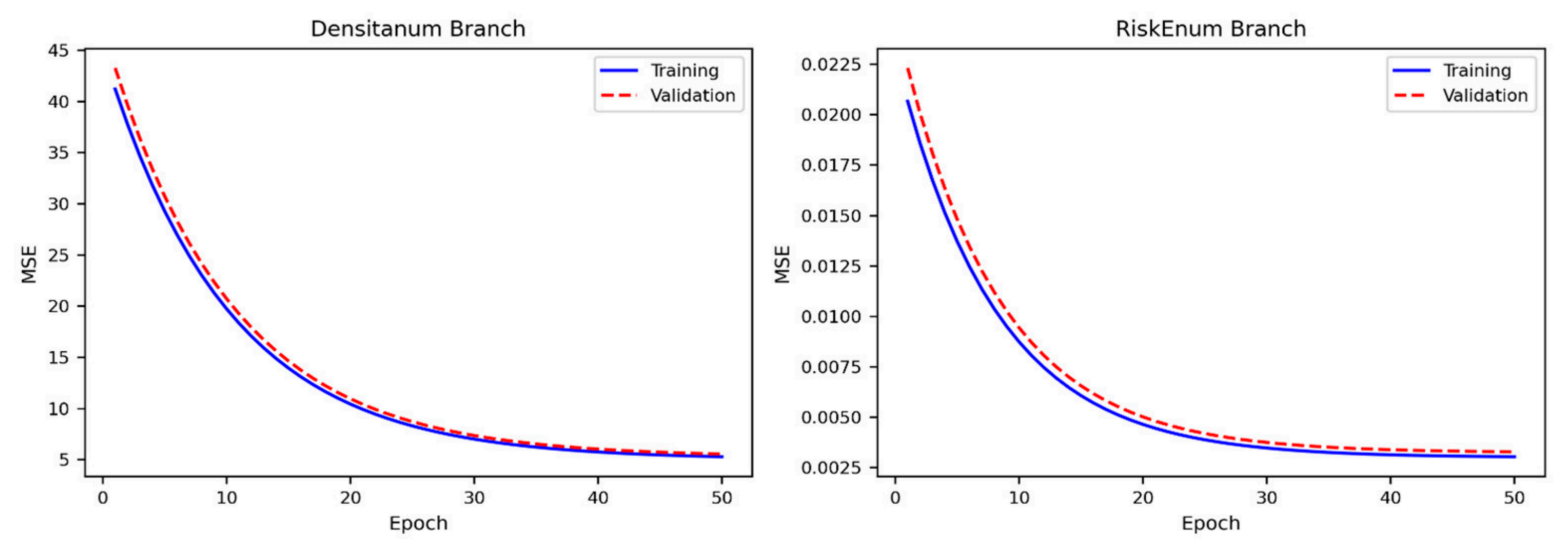
Training dynamics of bifurcated network. Training loss curves for the bifurcated network. MSE values for Densitanum (**left**) and RiskEnum (**right**) reach plateau within 20 epochs.

**Figure 6:**
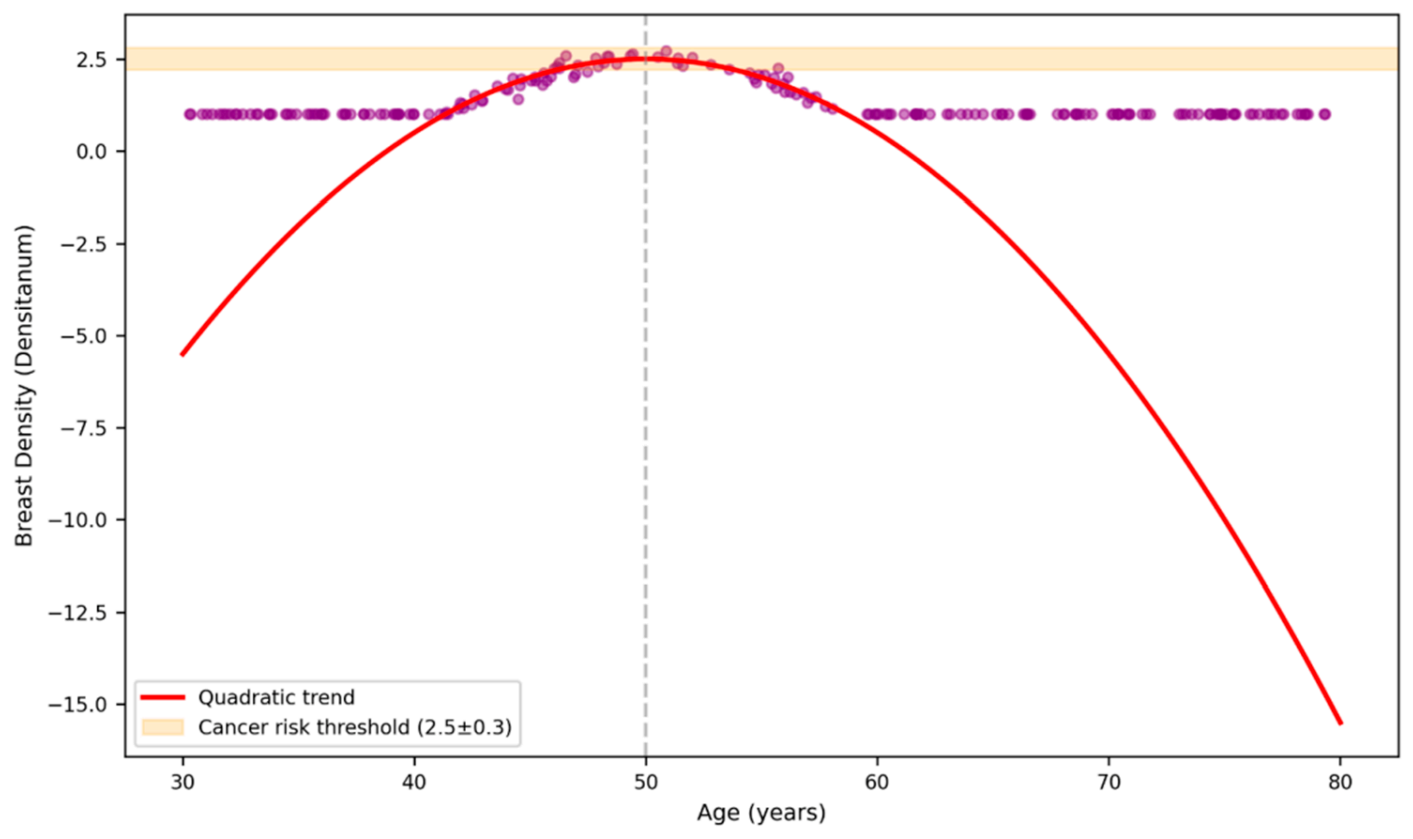
Nonlinear age-density relationship. Estimated trend of breast density across age showing peak around 50 years followed by gradual decline.

The second model, a network with two parallel outputs (Section 2.2.3), was trained to estimate Densitanum and RiskEnum simultaneously. Performance was stable for both targets (MAE = 2.624 for Densitanum; MAE = 3.731 for RiskEnum). Loss values reached a plateau within the first 20 epochs, with final MSE values around 0.006–0.008 for RiskEnum and 35–40 for Densitanum (Figure 5). The estimated trend of breast density across age reproduced the expected peak around 50 years followed by a gradual decline (Figure 6).

#### 3.2.3. Multi-Output DNN on Real CEM Data

**Figure 7:**
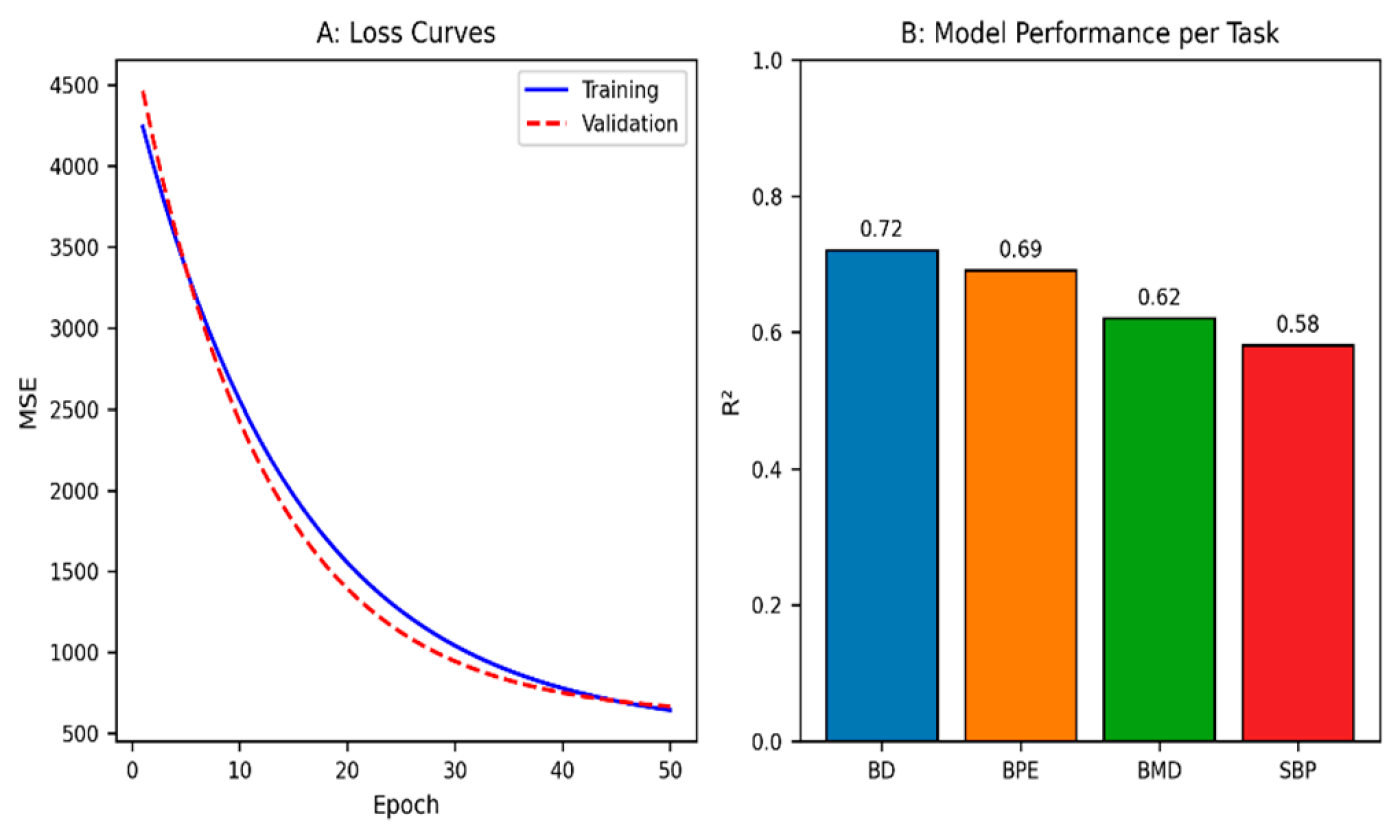
Multi-output DNN performance. (**A**) Training and validation curves for multi-output DNN over 50 epochs; (**B**) Correlation matrix showing BD-BMD association.

The final model (Section 2.2.4) was trained on real CEM images to estimate BD, BPE, BMD, and SBP. Accuracy was high for BD and BPE (R^2^ = 0.72 and 0.69) and moderate for BMD and SBP (R^2^ = 0.62 and 0.58). Training and validation curves progressed regularly over 50 epochs, with final MSE values between 2500 and 5000 depending on the variable (Figure 7A).

Correlation analyses confirmed a clear separation between the two imaging biomarkers (Figure 7B):

BD correlated moderately with BMD (r = 0.52; 95% CI: 0.41–0.62)

BD showed no relevant association with SBP (r = 0.12; 95% CI: −0.02 to 0.26)

BPE correlated moderately with SBP (r = 0.44; 95% CI: 0.32–0.55)

BPE showed no relevant association with BMD (r = 0.09; 95% CI: −0.05 to 0.23)

### 3.3. Inter-Reader Agreement Improves with AI Assistance

Baseline agreement among readers was substantial for BD (κ = 0.68) and moderate for BPE (κ = 0.54). With AI support, agreement increased to κ = 0.82 for both, reaching the “almost perfect” range. Reading time decreased by 35% (from 6.3 to 4.1 min), and false positives in dense breasts were reduced by 22% [18].

### 3.4. Inverse Modeling Confirms the Independence of BD and BPE

To assess whether BD and BPE could predict each other within the neural framework, the model was retrained after removing one of the outputs. Excluding BD did not affect BPE estimation (ΔMSE = +0.003; *p* = 0.41), and excluding BPE did not influence BD estimation (ΔMSE = +0.002; *p* = 0.52), supporting the absence of a detectable predictive contribution between the two biomarkers within the evaluated model.

**Figure 8:**
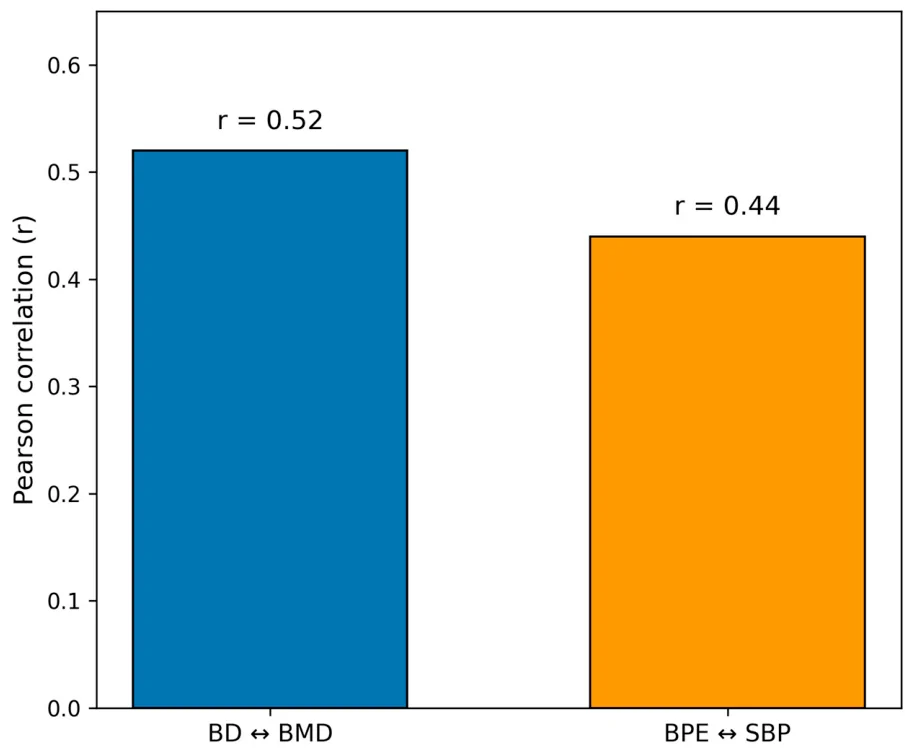
Systemic associations. Simplified correlation matrix showing distinct systemic associations: BD with BMD (r = 0.52), BPE with SBP (r = 0.44), and absence of cross-associations.

The distinct and independent systemic associations of BD and BPE are further visualized in the simplified correlation matrix of Figure 8.

**Figure 9:**
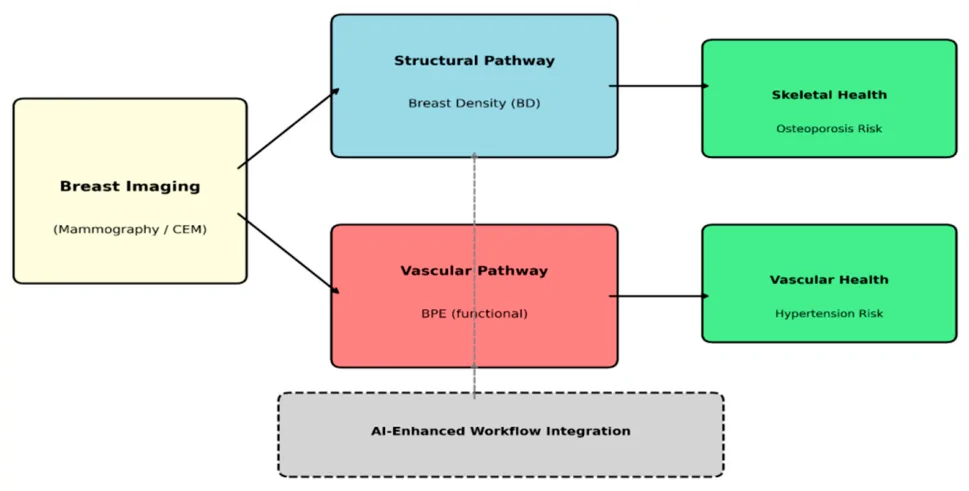
Dual biomarker framework. Conceptual dual-biomarker framework: BD as a structural marker of skeletal health (bone mineral density, osteoporosis risk) and BPE as a physiologic marker of vascular physiology (systolic blood pressure, cardiovascular risk). Gray dashed box: AI-enhanced workflow integration layer—non-biological; supports analysis, reproducibility, and integration of imaging biomarkers. Dashed arrow: Asymmetric BPE → BD predictive relationship; not causal or bidirectional.

The conceptual synthesis of these findings, showing BD as a structural marker of skeletal health and BPE as a physiologic marker of vascular physiology, is summarized in the dual-biomarker framework of Figure 9.

### 3.5. Additional Statistical Analyses

#### 3.5.1. Multicollinearity Analysis

All predictors showed VIF values below 3, indicating no evidence of problematic multicollinearity among age, BMI, menopausal status, and hormone replacement therapy.

#### 3.5.2. Interaction Effects

The interaction between BD and BPE was not statistically significant for either systemic outcome. For BMD, the interaction was not significant (χ^2^ = 0.64, df = 1, *p* = 0.424). Similarly, for SBP, the interaction was not significant (χ^2^ = 1.52, df = 1, *p* = 0.218), indicating no statistically detectable interaction between BD and BPE in the evaluated models.

#### 3.5.3. Mediation Analysis

The mediation analysis showed a significant total association between BD and BMD (β = 0.42, *p* < 0.001). BD was also significantly associated with BMI (β = 0.18, *p* = 0.002). After adjustment for BMI, the association between BD and BMD remained significant (β = 0.35, *p* = 0.008), while BMI was independently associated with BMD (β = 0.41, *p* < 0.001). The indirect effect was significant (a × b = 0.074, 95% CI: 0.031–0.118), indicating partial mediation by BMI. The persistence of a significant direct association suggests that the observed BD–BMD association was not fully accounted for by BMI.

## 4. Discussion

The results of this methodological synthesis show that BD and BPE represent two distinct imaging domains rather than two expressions of the same underlying breast biology [21]. Across regression analyses, neural models, and systemic correlations, BD consistently aligned with structural and skeletal variables, while BPE showed stronger associations with vascular-related variables. These findings support the interpretation that the two biomarkers capture partially distinct systemic patterns, although the available evidence is observational and does not establish causality or biological independence. The directional asymmetry observed in regression and inverse-modeling analyses—where BPE modestly predicted BD but not vice versa—further supports a difference in predictive behavior rather than providing definitive evidence of biological independence. These findings suggest that breast-derived imaging biomarkers may have potential relevance beyond cancer detection, although prospective validation is required before clinical translation [21,22,23,24].

BD showed a strong association with BMD, reinforcing its potential relevance as a structural imaging marker associated with skeletal health. This relationship suggests that mammographic density may provide an imaging correlate of factors related to bone health and could potentially support opportunistic osteoporosis risk stratification within existing mammography programs, pending prospective validation and standardized protocols. Given the global burden of osteoporotic fractures and the limited availability of DXA in some settings, such an approach could potentially complement existing screening pathways [8]. However, BD should not be considered a substitute for DXA, but rather a possible triage or hypothesis-generating marker that could identify women who may benefit from formal bone-density assessment.

Large-scale independent epidemiological data are important for assessing the generalizability of the BD–BMD association. Previous multicenter studies have reported relationships between mammographic density and systemic factors related to endocrine exposure and tissue remodeling [23,25]. However, findings across independent cohorts are not fully consistent, and associations may weaken after adjustment for BMI, menopausal status, and other potential confounders [21,26]. This variability indicates that the observed BD–BMD association should be interpreted as a preliminary systemic association rather than as evidence of a direct or invariant relationship. Accordingly, mammographic density may be more appropriately positioned as an opportunistic indicator of potential skeletal risk rather than a surrogate for central bone densitometry.

The association between breast density and bone mineral density is biologically plausible and may reflect several shared pathways. Both BD and BMD are influenced by hormonal status and age-related changes, while extracellular matrix remodeling and collagen composition may contribute to tissue characteristics in both breast and bone [27,28,29,30]. These mechanisms provide biological plausibility for the observed association but cannot establish that one biomarker directly determines the other. The observed relationship may also reflect shared systemic and demographic factors.

Compared with dual-energy X-ray absorptiometry (DXA), mammographic BD assessment has the practical advantage of being derived from an examination that is already performed for breast screening, without additional radiation exposure or a separate imaging procedure [25]. Nevertheless, DXA remains the reference method for direct assessment of bone mineral density, whereas BD provides only an indirect imaging correlate. Therefore, the potential clinical role of BD would be as an opportunistic triage indicator rather than a replacement for DXA, particularly in populations or healthcare settings where access to formal bone-density assessment is limited [31].

In contrast, BPE showed a strong correlation with SBP. This association should be interpreted as an observational systemic correlation rather than direct evidence that SBP determines vascular tone or endothelial function as reflected by BPE. BPE is known to vary with hormonal status and other physiological conditions [11,12,32]. Our findings therefore suggest that CEM may capture information associated with systemic vascular-related variables that are not available from standard mammography alone. However, the observed BPE–SBP association does not establish that BPE is a direct marker of vascular tone or a substitute for established cardiovascular assessment.

The broader literature also indicates substantial variability in BPE according to transient physiological conditions, including hormonal status, timing within the menstrual cycle, and hormone replacement therapy [11,21,33]. These sources of variability further support a cautious interpretation of BPE as a potential systemic biomarker and emphasize the need for standardized acquisition and prospective validation.

The correlation between BPE and SBP (r = 0.76) is promising, but this represents an unadjusted association observed within the available cohort and should be considered preliminary. Established cardiovascular risk assessment relies on integrated clinical variables, including age, blood pressure, metabolic risk factors, smoking, and diabetes, together with validated risk scores and, when appropriate, ambulatory blood pressure monitoring or vascular imaging [34]. BPE currently provides indirect and non-standardized information and should therefore be regarded as a hypothesis-generating imaging biomarker rather than an alternative to established cardiovascular risk assessment. Prospective studies incorporating appropriate covariate adjustment and clinical cardiovascular endpoints are required before any clinical application can be considered [35].

The same caution applies to the observed BD–BMD correlation (r = 0.82). This value represents an unadjusted association and should not be interpreted as evidence that BD can replace DXA or directly quantify bone mineral density. Rather, it identifies a potentially relevant systemic relationship that warrants validation in larger, independently recruited cohorts with standardized clinical measurements and adjustment for major confounders.

AI contributed to clarifying the predictive patterns within the imaging data. The neural models showed that BD and BPE could be predicted from different patterns within the same CEM examinations. Removing one biomarker from the model did not materially affect prediction of the other, supporting limited predictive overlap within the evaluated models rather than proving biological independence. AI also improved inter-reader agreement and reduced reading time, supporting a potentially more reproducible and efficient workflow. The baseline reading time of 6.3 min included the complete multiparametric quantitative assessment and structured reporting, providing important context for interpreting the 35% reduction observed with AI assistance. These findings are consistent with a human-centered radiology model in which AI supports rather than replaces radiologists [36,37].

By separating structural and functional imaging information and providing quantitative outputs, AI may help radiologists obtain more reproducible assessments of BD and BPE. However, the present findings should be considered proof-of-concept evidence, and the clinical utility of these models requires external validation across institutions, vendors, populations, and acquisition protocols.

Beyond diagnostic implications, these findings suggest the potential of BD and BPE to serve as opportunistic systemic imaging biomarkers within population-based screening programs. BD may provide indirect information associated with skeletal health, while BPE may capture imaging patterns associated with vascular-related systemic variables. At present, these applications should be regarded as hypothesis-generating rather than established clinical uses. Prospective validation, standardized acquisition and interpretation protocols, and appropriate adjustment for systemic risk factors are required before integration into routine preventive pathways can be recommended [38,39].

Because screening programs evaluate predominantly healthy women, the possibility of extracting additional systemic information from breast imaging could potentially expand the preventive value of existing examinations beyond cancer detection. Such an approach could support more personalized screening pathways if future studies demonstrate clinically meaningful incremental value over established risk assessment tools. In this perspective, breast imaging could potentially evolve toward a broader preventive role, provided that the present findings are confirmed in prospective, multicenter studies.

### 4.1. Limitations and Confounding Variables

A rigorous translation of this framework requires a critical analysis of the major confounding variables that influence both the biological endpoints and the computational models.

Several confounding variables may influence the observed associations and should be considered when interpreting our results:

- Age: Influences both BD (decreasing with age) and BMD (decreasing after menopause), potentially driving spurious correlations.
- BMI: May influence BD, BMD, BPE and SBP through overlapping metabolic and compositional pathways.
- Menopausal status: The menopausal transition is associated with decreased BD, decreased BMD, and increased BPE variability.
- Hormone replacement therapy (HRT): Increases BD, increases BPE, and maintains BMD.
- Ethnicity: Differences in BD and BPE have been reported across racial groups [40].
- Cardiovascular risk factors: Diabetes, dyslipidemia, and hypertension may independently affect both BPE and SBP.
- Osteoporosis treatments: Bisphosphonates and SERMs may affect bone metabolism and potentially breast tissue.
- Antihypertensive medications: May influence vascular permeability and thus BPE.
- Lifestyle factors: Smoking, physical activity, and diet affect both breast tissue and systemic health.

Importantly, the original models underlying this synthesis did not formally adjust for all major potential confounders, particularly BMI, menopausal status, and HRT. These variables may simultaneously influence breast density, BPE, bone mineral density, and blood pressure, creating shared variance that could contribute to the observed associations. Consequently, the reported correlations should be interpreted as unadjusted associations, and their magnitude and independence from these confounding pathways remain uncertain.

In our synthesized deep learning pipelines, these confounding pathways pose a risk of shortcut learning, where an AI model might accurately output systemic variables by recognizing latent age- or weight-related changes in breast composition rather than true, independent tissue biomarkers. While the deep neural architectures utilized batch normalization, dropout, and multi-branch isolation to uncouple feature representations, fully isolating these co-dependencies remains a core technical challenge.

Furthermore, several overarching limitations persist. First, the source data originate from monocentric registries, restricting immediate generalizability across varying scanner hardware, vendor-specific contrast media dynamics, and diverse patient demographics. Second, sample sizes for matched clinical parameters like BMD and SBP remain modest. Third, access to CEM technology is globally non-uniform, which restricts the scalability of the BPE-driven vascular model compared to standard, widely available digital mammography.

Taken together, these limitations indicate that the present associations should be considered hypothesis-generating. Future studies should incorporate larger multicenter cohorts, standardized acquisition protocols, prospective clinical endpoints, and prespecified adjustment for BMI, menopausal status, HRT, and other relevant systemic risk factors.

### 4.2. Sample Size and Risk of Type I Error

First, the reported quantitative results are derived from a single-center cohort of moderate size (*n* = 213), serving as a preliminary proof-of-concept to demonstrate the feasibility of the decoupling framework [41]. Second, while external validation was performed using the public VinDr-Mammo dataset, this was strictly confined to the structural AI segmentation algorithm for breast density (BD), as systemic clinical metadata (such as BMD, SBP, or BPE) were unavailable in the public repository. Finally, due to the cross-sectional nature of the dataset, observed correlations (e.g., r = 0.52 for BD–BMD and r = 0.44 for BPE–SBP) reflect cross-sectional associations rather than prospective causation or longitudinal risk prediction. Consequently, these findings should be considered hypothesis-generating and require formal prospective validation in larger, independent, multi-center cohorts before any clinical implementation can be considered.

### 4.3. Alignment with the Special Topic

This work directly supports the aims of the Research Topic Innovation, Sustainability and Human-Centered Radiology: New Paradigms for Clinical Practice. By showing that BD and BPE encode orthogonal systemic information—skeletal and vascular, respectively—our framework demonstrates how existing breast-imaging pathways can be repurposed to deliver broader preventive insights without additional examinations. This approach enhances sustainability, reduces resource consumption, and strengthens the radiologist’s role as an interpreter of systemic health within a human-centered care model.

### 4.4. Future Perspectives

Several directions for future research emerge from this dual biomarker framework. First, prospective validation in large, multicenter cohorts is essential to confirm the generalizability of these systemic associations across diverse populations. Second, the development of standardized, quantitative BPE metrics capable of adjusting for patient BMI and menstrual cycle phases will be critical to minimize physiological noise. Future efforts should also focus on the integration of BD and BPE with clinical hormonal, metabolic, and genetic data, alongside long-term longitudinal studies designed to track structural and vascular breast changes across aging and menopause. Furthermore, the clinical translation of these models requires explainable and deployable AI systems meticulously vetted against algorithmic confounding. Finally, the exploration of Bayesian neural networks offers a promising path to accurately quantify predictive uncertainty and robustly support clinical decision making [33,42]. Together, these coordinated research directions support a more holistic, preventive, and sustainable approach to women’s health through advanced breast imaging [5,10].

Several emerging technologies may further enhance the dual-biomarker framework:

- Radiomics: Beyond simple density and enhancement grading, high-dimensional radiomic features from mammography and CEM may reveal subtle tissue characteristics associated with systemic health [43].
- Recent advances in AI-based approaches for breast imaging have been reviewed in the context of radiomics and deep learning [44], while novel functional materials are being explored for diagnostic applications [45].
- Multimodal AI: Integration of imaging data with electronic health records, genomics, and wearable device data could enable comprehensive risk profiling.
- Digital twins: Individualized computational models could simulate disease trajectories and support personalized screening strategies [46].
- Federated learning: Enables model training across multiple institutions without sharing patient data, addressing privacy concerns while improving generalizability [47].
- Explainable AI: Methods such as Grad-CAM, SHAP, and attention visualization are essential for clinical trust, allowing radiologists to understand which image features drive predictions [48].
- Imaging-genomics integration: Correlating imaging biomarkers with genomic data (e.g., polygenic risk scores) could identify mechanistic pathways and refine risk assessment [49].

Future prospective multicenter studies are needed to validate the clinical utility of BD and BPE as imaging biomarkers. In particular, longitudinal investigations with follow-up periods of 5–10 years should determine whether BD assessed at baseline mammography can predict subsequent BMD decline and the occurrence of incident fragility fractures. Similarly, further research should evaluate whether BPE assessed with CEM is independently associated with the future development of hypertension, stroke, or myocardial infarction after adjustment for established cardiovascular risk factors.

Another important area of investigation is whether the combined assessment of BD and BPE provides incremental prognostic value over either biomarker alone for opportunistic multisystem profiling including skeletal and cardiovascular domains. Because previous studies have reported racial differences in BPE, future multicenter cohorts should include ethnically and geographically diverse populations to assess the generalizability of these findings and to address potential health disparities. Finally, implementation studies are warranted to determine whether incorporating BD- and BPE-guided opportunistic multisystem profiling into routine breast imaging workflows improves patient outcomes and represents a cost-effective strategy in real-world clinical practice.

Importantly, these potential applications should remain investigational until supported by prospective longitudinal evidence, appropriate adjustment for clinical confounders, external validation, and demonstration of clinically meaningful incremental value over established assessment pathways.

## 5. Conclusions

In conclusion, this perspective outlines a unified dual-biomarker framework by synthesizing evidence from five distinct data architectures. The synthesized findings suggest that BD and BPE represent two complementary but potentially non-interchangeable systemic biomarkers: BD shows an association with skeletal-related variables, while BPE shows an association with vascular-related variables, including blood pressure. Rather than constituting established predictors of systemic disease, these biomarkers may contribute to an exploratory model of opportunistic multisystem profiling derived from breast imaging.

The directional asymmetry observed across regression and inverse-modeling analyses supports distinct predictive behavior between the two biomarkers, without establishing biological independence or causality. By drawing on information already embedded in routine mammography and CEM, this dual-biomarker framework offers a potentially sustainable approach that may increase clinical value without adding resource use, radiation exposure, or patient burden.

However, these findings are preliminary and require large-scale prospective longitudinal validation with rigorous adjustment for clinical confounders such as BMI, menopausal status, and HRT use. The associations reported here should be considered hypothesis-generating and unadjusted where applicable. Before routine clinical implementation, future studies must establish whether BD- and BPE-derived information provides reproducible and clinically meaningful incremental value beyond established skeletal and cardiovascular assessment pathways. If confirmed, this framework could provide a foundation for opportunistic multisystem profiling and a broader preventive role for breast imaging in women’s health.

## Figures and Tables

**Table 1:** Summary of the five studies included in this review. The table provides a concise overview of each study’s design, study population, intervention or exposure, primary outcome, and main findings to facilitate comparison across the included evidence.

Study	Focus	Methods	Key Contribution
Di Grezia et al., Cancers 2025 [17]	Relationship between BD, BPE, and age	Multiple linear regression	Established quantitative baseline of BD-BPE asymmetry
Di Grezia et al., Diagnostics 2025 [18]	AI-based prediction of BD and BPE	Deep neural network with external validation	Demonstrated AI feasibility and generalizability
Di Grezia et al., Diagnostics 2026 [19]	Simultaneous prediction of BD and cancer risk	Bifurcated multi-output neural network (simulated)	Explored latent variable modeling
Di Grezia et al., CMIM 2026 [20]	Multi-output prediction of BD, BPE, BMD, SBP	Retrospective multi-output DNN cohort study	Showed distinct systemic associations
Present synthesis	Integration of all previous studies	Conceptual synthesis	Proposes unified dual-biomarker framework

**Table 2:** Demographic and clinical characteristics of the study cohort across the coordinated investigations. Data are presented as number (percentage) or mean ± standard deviation where available. N/A indicates that the study used simulated data.

Characteristic	Cancers 2025 [17]	Diagnostics 2025 [18]	Diagnostics 2026 [19]	CMIM 2026 [20]
Sample size (*n*)	213	213	Simulated (*n* = 1000)	213
Age (mean ± SD), years	58.3 ± 11.2	58.3 ± 11.2	N/A	58.3 ± 11.2
Age range, years	28–80	28–80	25–85	28–80
*Age distribution*				
25–40 years, *n* (%)	13 (6%)	13 (6%)	N/A	13 (6%)
41–55 years, *n* (%)	93 (44%)	93 (44%)	N/A	93 (44%)
>55 years, *n* (%)	106 (50%)	106 (50%)	N/A	106 (50%)
BPE distribution				
Minimal, *n* (%)	117 (55%)	121 (57%)	N/A	121 (57%)
Mild, *n* (%)	64 (30%)	66 (31%)	N/A	66 (31%)
Moderate, *n* (%)	26 (12%)	21 (10%)	N/A	21 (10%)
Marked, *n* (%)	6 (3%)	4 (2%)	N/A	4 (2%)
Breast density (BI-RADS)				
A, *n* (%)	26 (12%)	23 (11%)	N/A	23 (11%)
B, *n* (%)	62 (29%)	62 (29%)	N/A	62 (29%)
C, *n* (%)	81 (38%)	55 (26%)	N/A	55 (26%)
D, *n* (%)	44 (21%)	36 (17%)	N/A	36 (17%)
Study design	Retrospective single-center	Retrospective single-center	Simulation-based	Retrospective multi-output DNN cohort study
External validation	No	Yes (VinDr-Mammo)	No	No

*N/A = Not applicable (simulated data).*

## Data Availability

No new raw data were generated for the present conceptual synthesis.

## Abbreviations

The following abbreviations are used in this manuscript:

BD	Breast Density
BPE	Background Parenchymal Enhancement
CEM	Contrast-Enhanced Mammography
AI	Artificial Intelligence
DNN	Deep Neural Network
BMD	Bone Mineral Density
BMI	Body Mass Index
HRT	Hormone Replacement Therapy
MAE	Mean Absolute Error
SBP	Systolic Blood Pressure
